# IGF‐1R pathway activation as putative biomarker for linsitinib therapy to revert tamoxifen resistance in ER‐positive breast cancer

**DOI:** 10.1002/ijc.32668

**Published:** 2019-10-06

**Authors:** Dinja T. Kruger, Xanthippi Alexi, Mark Opdam, Karianne Schuurman, Leonie Voorwerk, Joyce Sanders, Vincent van der Noort, Epie Boven, Wilbert Zwart, Sabine C. Linn

**Affiliations:** ^1^ Department of Medical Oncology Amsterdam UMC, Vrije Universiteit Amsterdam/Cancer Center Amsterdam Amsterdam The Netherlands; ^2^ Division of Molecular Pathology The Netherlands Cancer Institute Amsterdam The Netherlands; ^3^ Division of Oncogenomics Oncode Institute, The Netherlands Cancer Institute Amsterdam The Netherlands; ^4^ Division of Molecular Oncology & Immunology The Netherlands Cancer Institute Amsterdam The Netherlands; ^5^ Department of Pathology The Netherlands Cancer Institute Amsterdam The Netherlands; ^6^ Division of Biometrics The Netherlands Cancer Institute Amsterdam The Netherlands; ^7^ Department of Biomedical Engineering Eindhoven University of Technology Eindhoven The Netherlands; ^8^ Department of Medical Oncology The National Cancer Institute Amsterdam The Netherlands; ^9^ Department of Pathology University Medical Center Utrecht Utrecht The Netherlands

**Keywords:** breast cancer, IGF‐1 receptor, PI3K/MAPK pathway, adjuvant tamoxifen, linsitinib

## Abstract

Preclinical studies indicate that activated IGF‐1R can drive endocrine resistance in ER‐positive (ER+) breast cancer, but its clinical relevance is unknown. We studied the effect of IGF‐1R signaling on tamoxifen benefit in patients and we searched for approaches to overcome IGF‐1R‐mediated tamoxifen failure in cell lines. Primary tumor blocks from postmenopausal ER+ breast cancer patients randomized between adjuvant tamoxifen *versus* nil were recollected. Immunohistochemistry for IGF‐1R, p‐IGF‐1R/InsR, p‐ERα(Ser118), p‐ERα(Ser167) and PI3K/MAPK pathway proteins was performed. Multivariate Cox models were employed to assess tamoxifen efficacy. The association between p‐IGF‐1R/InsR and PI3K/MAPK pathway activation in MCF‐7 and T47D cells was analyzed with Western blots. Cell proliferation experiments were performed under various growth‐stimulating and ‐inhibiting conditions. Patients with ER+, IGF‐1R‐positive breast cancer without p‐IGF‐1R/InsR staining (*n* = 242) had tamoxifen benefit (HR 0.41, *p* = 0.0038), while the results for p‐IGF‐1R/InsR‐positive patients (*n* = 125) were not significant (HR 0.95, *p* = 0.3). High p‐ERα(Ser118) or p‐ERα(Ser167) expression was associated with less tamoxifen benefit. In MCF‐7 cells, IGF‐1R stimulation increased phosphorylation of PI3K/MAPK proteins and ERα(Ser167) regardless of IGF‐1R overexpression. This could be abrogated by the dual IGF‐1R/InsR inhibitor linsitinib, but not by the IGF‐IR‐selective antibody 1H7. In MCF‐7 and T47D cells, stimulation of the IGF‐1R/InsR pathway resulted in cell proliferation regardless of tamoxifen. Abrogation of cell growth was regained by addition of linsitinib. In conclusion, p‐IGF‐1R/InsR positivity in ER+ breast cancer is associated with reduced benefit from adjuvant tamoxifen in postmenopausal patients. In cell lines, stimulation rather than overexpression of IGF‐1R is driving tamoxifen resistance to be abrogated by linsitinib.

## Abstract 2

**What's new?**

In breast cancer, the growth factor receptor IGF‐1R is frequently overexpressed, boosting cell proliferation and transformation. Prior work has shown that IGF‐1R pathway activation promotes treatment resistance in cell lines. Here, the authors evaluated whether the pathway contributes to tamoxifen resistance in patients. In patients with ER+ breast cancer, patients whose cancers tested negative for activated IGF‐R1 benefited from adjuvant tamoxifen, while those with cancers tested positive showed little improvement. Next, they showed that treating breast cancer cells with linsitinib can thwart IGF‐1R signaling and restore tamoxifen's efficacy. Patients with activated IGF‐1R pathways may therefore do better with tamoxifen combined with linsitinib than tamoxifen alone.

**Abbreviations**
AI: aromatase inhibitor
ER+: ERα‐positive
ERα: estrogen receptor alpha
FFPE: formalin‐fixed paraffin‐embedded
HER2−: human epidermal growth factor receptor 2‐negative
HR: hazard ratio
IGF‐1R: insulin‐like growth factor‐1 receptor β
IKA: Integraal Kankercentrum Amsterdam
InsR: insulin receptor
MAPK: mitogen‐activated protein kinase
PI3K: phosphatidylinositol 3‐kinase
p‐IGF‐1R: phosphorylated IGF‐1R
PR: progesterone receptor
RFI: recurrence‐free interval
TMAs: tissue microarrays

## Introduction

Approximately 70% of breast cancer cases express estrogen receptor alpha (ERα). For patients with primary ER‐positive (ER+) breast cancer, adjuvant endocrine therapy improves recurrence‐free and overall survival.^1^, ^2^ Current guidelines for postmenopausal patients include antiestrogens, such as tamoxifen, aromatase inhibitors (AIs) or a sequential treatment of the two drugs.^2^, ^3^ Unfortunately, patients treated for ER+ disease still have a significant recurrence risk during extended follow‐up.^4^ Therefore, research efforts are being undertaken to elucidate mechanisms of endocrine resistance.^5^

Molecular mechanisms of endocrine treatment resistance include overexpression or amplification of growth factor receptors, downstream activation of the phosphatidylinositol 3‐kinase (PI3K)/Akt/mammalian target of rapamycin or mitogen‐activated protein kinase (MAPK) signaling pathway and alterations in ERα phosphorylation status.^6^, ^7^, ^8^, ^9^ One of the growth factor receptors frequently (over)expressed in primary ER+ breast cancer is the insulin‐like growth factor‐1 receptor β (IGF‐1R) with percentages varying between 53.8% and 80%.^10^, ^11^ When IGF‐1R becomes activated (phosphorylated IGF‐1R [p‐IGF‐1R]) after binding its ligands IGF Type 1, 2 or insulin, this transmembrane tyrosine kinase receptor can activate downstream signaling of the PI3K and MAPK pathways to promote cell proliferation, differentiation, survival as well as transformation.^7^, ^12^ These pathways are also known to induce ERα phosphorylation at ERα(Ser118) (MAPK pathway)^13^, ^14^ and ERα(Ser167) (PI3K pathway).^15^ The phosphorylation status of these two ERα residues has previously been described to associate with breast cancer patient outcome.^16^, ^17^ Furthermore, the IGF‐1R receptor shares high homology with the insulin receptor (InsR) with which it can heterodimerize.^18^, ^19^

Little clinical evidence is available on the role of IGF‐1R expression in endocrine resistance for ER+ breast cancer. Our group previously demonstrated in ER+/human epidermal growth factor receptor 2‐negative(HER2−) breast cancer patients that total levels of IGF‐1R did not predict resistance to adjuvant tamoxifen.^20^ In a small study on invasive primary and recurrent tamoxifen‐resistant tumors of the same patient, there was no evidence of increased IGF‐1R expression.^21^ Whether IGF‐1R phosphorylation and concurrent activation of the PI3K and/or MAPK pathway are indicative for response to adjuvant endocrine therapy in breast cancer patients remains elusive.

In cell lines, the direct link between IGF‐1R expression and endocrine response has been studied more extensively. Exogenous introduction of IGF‐1R in MCF‐7 breast cancer cells activated PI3K and MAPK pathways, rendered the cells less dependent on ERα signaling and consequently less responsive to tamoxifen as well as to the full ERα‐antagonist fulvestrant.^22^ Proliferation of long‐term estrogen‐deprived MCF‐7 cells was reported to rely on increased p‐IGF‐1R expression and subsequent PI3K pathway signaling.^23^ Furthermore, IGF‐1R phosphorylation, but not increased IGF‐1R protein expression, was found to be associated with tamoxifen resistance in MCF‐7 xenografts.^24^

Cumulatively, while cell line studies show that activation of the IGF‐1R pathway is able to drive endocrine resistance, this phenomenon has not yet been studied in a clinical data set. We here reveal the predictive value of positive p‐IGF‐1R/InsR expression for diminished adjuvant tamoxifen benefit in a large cohort of primary ER+ breast cancer patients. Experiments in ER+ breast cancer cell lines demonstrate that activation of the IGF‐1R signaling pathway is associated with PI3K/MAPK pathway signaling, p‐ERα(Ser167) phosphorylation and cell proliferation in the presence of tamoxifen. Finally, we show the potential usefulness of the dual IGF‐1R/InsR inhibitor linsitinib to overcome tamoxifen resistance in IGF‐1R‐driven ER+ breast cancer.

## Materials and Methods

### Patients and material

Formalin‐fixed paraffin‐embedded (FFPE) primary tumor tissue blocks were recollected from patients who participated in the Integraal Kankercentrum Amsterdam (IKA) trial (July 1982 until September 1993).^8^ Tumor specimens were fixed in buffered formalin according to the manufacturer's protocol and to local hospital guidelines. Data from this trial have been incorporated in the Early Breast Cancer Trialists' Collaborative Group meta‐analysis on the efficacy of adjuvant tamoxifen in primary breast cancer.^1^ In the IKA trial, 1,662 postmenopausal patients with Stages I–III breast cancer of any subtype were recruited and were randomized (2:1) between adjuvant tamoxifen (30 mg/day) for 1 year *versus* no adjuvant therapy. After 1 year, patients on tamoxifen were randomized a second time to continue tamoxifen for another 2 years or to stop further treatment. In 1988, two interim analyses demonstrated a significant improvement in recurrence‐free survival in lymph node‐positive patients who received tamoxifen. After these analyses, all node‐positive patients skipped the first randomization and were given 1 year of tamoxifen before participating in the second randomization. None of the patients received adjuvant chemotherapy. The IKA trial was approved by the central ethics committee of the Netherlands Cancer Institute and all patients had provided informed consent before participation.

Primary tumor material could be obtained from 739 patients for later studies. Patient characteristics did not differ as compared to the original study population.^8^, ^20^ For these retrospective studies, no additional consent was required according to Dutch legislation,^25^ since the use of archival pathology left‐over material does not interfere with patient care. Tumor material was handled according to the Dutch code of conduct for dealing responsibly with human tissue in the context of health research.^26^

### Immunohistochemistry

Tissue microarrays (TMAs) were constructed from the FFPE blocks using three 0.6 mm cores. The TMAs were stained for ERα, progesterone receptor (PR) and HER2. ERα and PR were considered positive if ≥1% of tumor cells exhibited nuclear staining. Additional analyses with a cutoff of ≥10% according to the Dutch breast cancer guideline were also performed. HER2 was considered positive if membranous staining was DAKO score 3, implying that >10% of the tumor cells exhibited strong circumferential staining. In the case of DAKO score 2, for example, ≤10% of the tumor cells exhibited strong circumferential staining or >10% of the tumor cells exhibited medium or incomplete staining, chromogenic *in situ* hybridization was performed. HER2 was considered amplified if more than six copies of the gene were found. Tumor grade was scored on a hematoxylin–eosin stained slide according to the modified Bloom–Richardson scoring system.^27^ Both tumor grade and histological subtype were revised by a pathologist. Methodology for staining of IGF‐1R, PTEN, p‐Akt(Thr308), p‐Akt(Ser473), p‐4EBP1, p‐p70S6K, p‐MAPK and p‐S6RP has been reported elsewhere^8^, ^20^, ^28^ (Table S1). Immunohistochemistry data of the same patients has previously been used to generate a classification tool to distinguish tumors with predominant positive or negative PI3K/MAPK pathway activation.^28^

**Figure 1:**
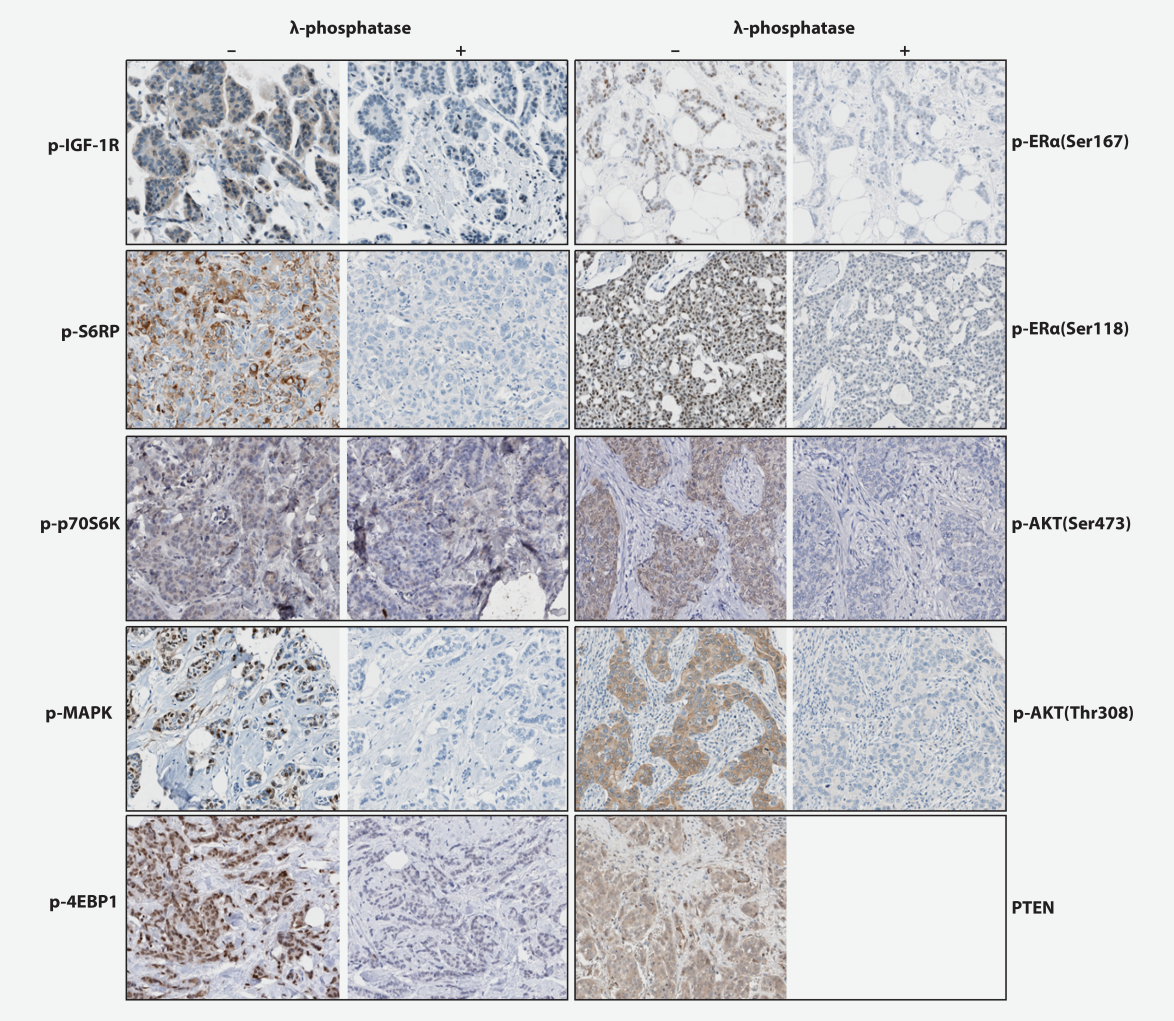
Representative immunohistochemistry images. Representative immunostaining pictures. For p‐IGF‐1R, p‐p70S6K, p‐AKT473, p‐4EBP1, p‐ERα(Ser167), p‐ERα(Ser118), p‐MAPK, p‐AKT308 and p‐S6RP, the left panels beneath “–” represents positive TMA cores without previous λ‐phosphatase treatment. The right panels beneath “+” represents positive TMA cores after λ‐phosphatase treatment resulting in negative staining. Since PTEN was not a phospho‐staining, no λ‐phosphatase treatment was performed and only a positive TMA core (left panel) was shown. [Color figure can be viewed at http://wileyonlinelibrary.com]

In the present study, immunohistochemistry was carried out for p‐IGF‐1R(Tyr1131)/InsR (Cell Signaling #3021, Danvers, MA), p‐ERα(Ser118) (Cell Signaling #2511) and p‐ERα(Ser167) (Cell Signaling #5587) (Table S1). The antibodies were submitted to several validation tests in addition to those performed by the manufacturer. Staining protocols from the manufacturer were tested with several dilutions. Furthermore, the antibodies were tested on a variety of (tumor) tissues to check positive or negative staining. Phospho‐specificity of the respective antibodies was validated by λ‐phosphatase pretreatment, resulting in signal loss (Fig. 1). All tests were assessed by the pathologist for approval and implementation. Representative immunohistochemistry pictures are shown in Figure 1. Staining for p‐ERα(Ser118) and p‐ERα(Ser167) was performed using a standardized protocol on the Ventana Benchmark® Ultra system (Ventana Medical Systems). For p‐IGF‐1R/InsR, antigen retrieval was performed using EDTA buffer and slides were incubated for 36 min with antibody (dilution 1:100). Age of the tumor samples and different fixation procedures did not affect the phosphoprotein staining procedure.^8^

After activation, the IGF‐1 receptor is internalized,^29^ so cytoplasmic intensity (0–3) was assessed for p‐IGF‐1R/InsR. Since the p‐IGF‐1R/InsR antibody also detects InsR, only the p‐IGF‐1R/InsR scores of patients positive for IGF‐1R (membranous score 1–3) was used to increase the specificity of p‐IGF‐1R detection. Correlation plots between IGF‐1R, membranous p‐IGF‐1R/InsR and cytoplasmic p‐IGF‐1R/InsR are presented in the supplements (Fig. S1). For both p‐ERα(Ser118) and p‐ERα(Ser167), the percentage of positive nuclei was calculated. Two TMAs were assessed in a blinded manner by a second observer and these scorings were analyzed as binary factor to calculate the interobserver variability expressed as kappa coefficient (Table S1).^30^ For further analyses, we used the scores generated by one of the two observers (M.O.). The highest score out of three cores from the same tumor was used for the statistical analyses for all antibodies. For p‐IGF‐1R/InsR, samples were dichotomized into negative *versus* positive staining. For p‐ERα(Ser118) and p‐ERα(Ser167) we used the median of 50% as cutoff, which was further affirmed by the binary aspect of bar plots from the scorings (Fig. S2).

### Breast cancer cell lines and cell proliferation

MCF‐7 and T47D human breast cancer cell lines obtained from American Type Culture Collection (Manassas, VA) were authenticated by short tandem repeat profiling. MCF‐7 cells were cultured in Dulbecco's Modified Eagle's Medium (DMEM, 11880‐028, Gibco, Thermo Fisher Scientific, Waltham, MA), supplemented with 2 mM L‐glutamine (200 mM L‐Glutamine, 25030‐081, Gibco, Thermo Fisher Scientific) and 10% Fetal Bovine Serum (FBS, F7524, Sigma‐Aldrich, Saint Louis, MO). T47D cells were cultured in RPMI 1640 medium (11875‐093, Gibco), supplemented with 10% FBS and 10 μg/ml insulin (Sigma‐Aldrich). To generate MCF‐7 cells overexpressing IGF‐1R and control cells, FuGENE 6‐mediated transfection of, respectively, pcDNA1‐IGF‐1R or pcDNA1‐empty vector that both contained a *neomycin* resistance gene was performed. After 2 days, cells were treated with neomycin (Bio‐Connect, Huissen, NL) for 14 days after which the surviving cells were maintained in the presence of 0.2 mg/ml neomycin. Overexpression of IGF‐1R was quantified after reloading a subset of the samples of Figure 3 on a new single Western blot (Fig. S3). Average pixel intensity for pIGF‐1R normalized for actin and corrected for the background signal demonstrated an overexpression:control ratio of 5.9 (SD 2.2).

Prior to all cell proliferation experiments, cells were cultured 72 hr in hormone‐deprived 5% charcoal/dextran‐treated FBS (HyClone™, GE Healthcare Life Sciences, Little Chalfont, United Kingdom) after which they were seeded into 384‐wells plates. Cells were then cultured in media containing 0.1 nM estradiol and 10 nM 4‐hydroxytamoxifen (Sigma‐Aldrich) (further referred to as tamoxifen or 4‐OHT) or 10 nM fulvestrant (Tocris Bioscience, Bristol, United Kingdom). These conditions were combined with 100 ng/ml IGF‐1 (Peprotech, Rocky Hill, NJ), 100 ng/ml IGF‐2 (Peprotech), 10 ug/ml insulin (Sigma‐Aldrich) or DMSO vehicle control. Furthermore, all conditions were cultured with or without linsitinib, a dual IGF‐1R/InsR inhibitor (Selleck Chem, Houston, TX), or 1H7, a selective antibody against IGF‐1R (Thermo Fischer Scientific). Linsitinib was added at 1 μM final concentration for MCF‐7 cells and 20 μM for T47D cells. 1H7 was added at a concentration of 0.3 μg/ml 1H7.^31^ As additional control experiments, estradiol alone or DMSO vehicle control were included. All conditions were tested in triplicate or quadruplicate, and two independent biological replicates. For a period of 2 weeks, the confluence of each well was continuously assessed using IncuCyte ZOOM® technology and software (Essen Bioscience, Welwyn Garden City, United Kingdom).

Mycoplasma testing was performed in‐house by the MycoAlert™ mycoplasma detection kit (Lonza, Bazel, Switzerland). All cultured cells were found to be mycoplasma negative.

### Western blot

Cells were cultured 72 hr in hormone‐deprived 5% charcoal/dextran‐treated FBS. Cells were preincubated with inhibitors (1H7 or linsitinib, where indicated) for 30 min, and subsequently stimulated for 10 min with IGF‐1, IGF‐2 or insulin in the absence or presence of (anti)estrogen. Cells were then washed once with ice‐cold PBS and harvested in 2x Laemmli buffer supplemented with protease and phosphatase inhibitors. Total protein content was quantified by using the BCA assay (23227, Thermo Fisher Scientific). Cell lysates containing equal amounts of protein were analyzed by SDS‐PAGE and Western blot. Antibodies against IGF‐1R, p‐IGF‐1R/InsR(Tyr1135/1136), InsR‐β, p‐ERα(Ser118), p‐ERα(Ser167), ERα, p‐Akt(Thr308), p‐Akt(Ser473), Akt1, p‐p44/42(Thr202/Tyr204)MAPK(ERK1/2) and MAPK were used and actin served as control (Table S2).

### Statistics

Recurrence‐free interval (RFI) was defined as the time from the first randomization to the occurrence of a local, regional or distant recurrence or breast cancer‐specific death.^32^ Patients with a secondary contralateral breast tumor were censored at the time of the contralateral diagnosis, since it was not possible to link breast cancer‐specific events to the first or to the contralateral malignancy. Patients who died from other causes or who were lost to follow‐up were censored at the time of this occurrence.

Fisher's exact test was used to test a potential association between (phosphorylated) protein expression in breast cancer tissue and clinicopathological factors. After stratification for lymph node status, multivariate Cox models were employed to assess hazard ratios (HRs) of RFI for respectively p‐IGF‐1R/InsR, p‐ERα(Ser118) and p‐ERα(Ser167) expression and adjuvant tamoxifen followed by testing a possible interaction between tamoxifen treatment and each of the four proteins. Covariates included in the multivariate tests were age (<65 *vs*. ≥65), histological grade (grade 3 *vs*. grades 1 to 2), tumor size (T3 to T4 *vs*. T1 to T2), histological subtype (lobular *vs*. ductal), PR status (negative *vs*. positive) and HER2 status (negative *vs*. positive). The prognostic potential of markers was analyzed using data from patients assigned to the control group, but estimating the baseline hazard using data from both tamoxifen and control patients. All survival analyses were stratified for lymph node status and corrected for the above‐described covariates. The Kaplan–Meier method was used to create RFI curves. Previously, we have developed a classification tool to distinguish ER+, HER2‐negative breast cancer patients either with and or without preferential PI3K/MAPK pathway activation.^28^ This tool was used in the current setting.

IBM SPSS statistics (Windows version 22) and R for statistics (Windows version 3.3.1) were used for the statistical analyses. A *p* value of <0.05 was considered statistically significant.

## Results

### Association of p‐IGF‐1R/InsR with clinicopathological characteristics, tamoxifen benefit and prognosis

From a total of 739 tumors, 566 were positive for ERα. In this population, 443 cases could be assessed for IGF‐1R of which 393 scored positive and 50 were negative. Since we hypothesized that phosphorylation of IGF‐1R would be predictive for tamoxifen resistance, we only selected tumor samples harboring IGF‐1R expression to evaluate the p‐IGF‐1R/InsR scoring. In the IGF‐1R‐positive population, 367 cases were available to assess p‐IGF‐1R/InsR levels (Table 1). In this group, 90 RFI events occurred and the median follow‐up of patients without an event was 8.3 years (7.9–8.9). Reasons why cases could not be included were TMAs without residual invasive tumor, with ductal carcinoma *in situ* or cores with technical errors. Patients with tumors positive for p‐IGF‐1R had more often PR‐positive tumors (Table 2).

**Table 1:** Distribution of clinicopathological characteristics of patients with a tumor available for p‐IGF‐1R/IR staining derived from the ER+ population and that of the total IKA trial population

		p‐IGF‐1R/IR population *N* (%)	ER+ population *N* (%)	Total study population *N* (%)
Total		367 (100)	566 (100)	1,662 (100)
Age	<65	172 (47)	270 (48)	869 (52)
	≥65	195 (53)	296 (52)	793 (48)
Lymph node status	Negative	200 (55)	311 (55)	901 (54)
	Positive	167 (45)	255 (45)	761 (46)
T stage	T1–T2	322 (88)	506 (89)	1,482 (89)
	T3–T4	45 (12)	60 (11)	180 (11)
Grade	Grades 1–2	231 (63)	375 (67)	435 (59)
	Grade 3	136 (37)	191 (33)	304 (41)
Histological subtype^1^	Ductal	282 (77)	405 (72)	540 (32)
	Lobular	25 (7)	59 (10)	66 (4)
	Ductolobular	21 (6)	30 (5)	32 (2)
	Mucinous	8 (2)	14 (3)	16 (1)
	Metaplastic	1 (0)	1 (0)	5 (0.3)
	Medullary	1 (0)	1 (0)	7 (0.4)
	Tubulolobular	3 (1)	7 (1)	7 (0.4)
	Other	10 (3)	20 (4)	28 (2)
	Missing	16 (4)	29 (5)	961 (58)
HER2 status	Negative	329 (90)	489 (86)	594 (36)
	Positive	33 (9)	44 (8)	85 (5)
	Missing	5 (1)	33 (6)	983 (59)
PR status	Negative	130 (36)	204 (36)	346 (21)^2^
	Positive	236 (64)	345 (61)	513 (31)^2^
	Missing	1 (0)	17 (3)	803 (48)
ER status	Negative	0 (0)	0 (0)	311 (24)^2^
	Positive	367 (100)	566 (100)	1,014 (77)^2^

1 Only revised scorings are shown of available tumors from 739 patients.

2 Determined by ligand‐binding assay in the original IKA trial.

**Table 2:** Association between clinicopathological factors and downstream proteins in tumors scoring negative or positive for p‐IGF‐1R/IR in IGF‐1R positive breast tumors

		p‐IGF‐1R/IR		
		Negative	Positive	
		*N* (%)	*N* (%)	*p*‐Value*
Age	<65	109 (45)	63 (50)	0.38
	≥65	133 (55)	62 (50)	
Lymph node status	Negative	131 (54)	69 (55)	0.91
	Positive	111 (46)	56 (45)	
T stage	T1–T2	209 (86)	113 (90)	0.32
	T3–T4	33 (14)	12 (10)	
Grade	Grades 1–2	158 (65)	73 (58)	0.21
	Grade 3	84 (35)	52 (42)	
Histological subtype	Ductal	188 (90)	94 (95)	0.26
	Lobular	20 (10)	5 (5)	
HER2 status	Negative	220 (92)	109 (88)	0.18
	Positive	18 (8)	15 (12)	
PR status	Negative	95 (49)	35 (41)	0.039
	Positive	147 (61)	89 (72)	
PTEN	0	47 (21)	7 (6)	0.00016
	1–3	181 (79)	114 (94)	
p‐Akt(Thr308)	0	160 (69)	32 (27)	<0.0001
	1–3	72 (31)	88 (73)	
p‐Akt(Ser473)	0–1	125 (58)	15 (13)	<0.0001
	2–3	91 (42)	100 (87)	
p‐4EBP1	0–20%	112 (49)	21 (18)	<0.0001
	30–100%	117 (51)	97 (82)	
p‐p70S6K	0	118 (51)	17 (14)	<0.0001
	1–3	112 (49)	101 (86)	
p‐MAPK	0%	123 (55)	18 (15)	<0.0001
	10–100%	102 (45)	103 (85)	
p‐S6RP	0–10%	92 (40)	21 (18)	<0.0001
	20–100%	139 (60)	95 (82)	
p‐ERα(Ser118)	0–40%	168 (73)	30 (25)	<0.0001
	50–100%	62 (27)	88 (75)	
p‐ERα(Ser167)	0–40%	194 (82)	55 (45)	<0.0001
	50–100%	43 (18)	67 (55)	

* Fisher's exact test based on cases without missing values.

**Figure 2:**
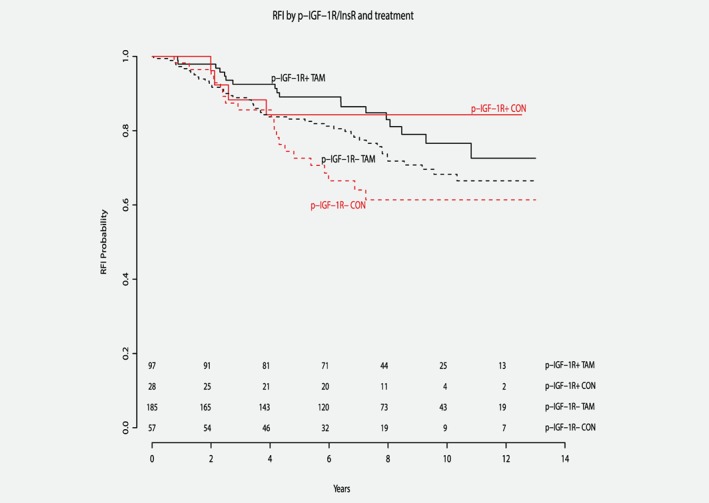
Lack of p‐IGF‐1R/InsR expression is associated with tamoxifen benefit. Kaplan–Meier curves for recurrence‐free interval according to tamoxifen treatment and p‐IGF‐1R/InsR status in ER+ patients. Multivariate *p* for interaction = 0.23. Abbreviations: TAM, patients treated with tamoxifen; CON, control patients not treated with tamoxifen; p‐IGF‐1R+, patients with p‐IGF‐1R/InsR positive tumors; p‐IGF‐1R‐, patients with p‐IGF‐1R/InsR negative tumors; RFI, recurrence‐free interval. [Color figure can be viewed at http://wileyonlinelibrary.com]

Patients without p‐IGF‐1R/InsR staining (*n* = 242) derived significant tamoxifen benefit (multivariate HR 0.41, 95% confidence interval [CI] 0.22–0.75, *p* = 0.0038), while the results for p‐IGF‐1R/InsR‐positive patients (*n* = 125) were not significant (multivariate HR 0.95, 95% CI 0.27–3.38, *p* = 0.34; Fig. 2 and Table 3). The interaction test of p‐IGF‐1R/InsR with tamoxifen benefit was not significant (multivariate *p* for interaction = 0.23). Positivity of p‐IGF‐1R/InsR was significantly associated with an improved RFI, pointing toward a possible prognostic role (multivariate HR = 0.27, 95% CI 0.08–0.95, *p =* 0.041; Table 3). When the analyses were performed with ER and PR cutoffs of 10%, the results did not substantially change (data not shown).

**Table 3:** Multivariate Cox proportional hazard model of recurrence‐free interval including p‐IGF‐1R/IR status and treatment interaction

Variable		HR	95% CI	*p*
Interaction	p‐IGF‐1R/IR with treatment			0.23
Tamoxifen *vs*. CON	p‐IGF‐1R/IR‐negative group	0.41	0.22–0.75	0.0038
	p‐IGF‐1R/IR‐positive group	0.95	0.27–3.38	0.34
p‐IGF‐1R/IR positive *vs*. negative	CON patients	0.27	0.08–0.95	0.041
Age	≥65 *vs*. <65 (ref)	1.01	0.64–1.61	0.97
T stage	T3‐4 *vs*. T1‐2 (ref)	1.22	0.67–2.22	0.52
Grade	Grade 3 *vs*. grades 1–2 (ref)	1.48	0.89–2.44	0.13
Histological subtype	Lobular *vs*. ductal (ref)	2.69	1.35–5.37	0.0049
HER2 status	Positive *vs*. negative (ref)	1.42	0.69–2.93	0.35
PR status	Positive *vs*. negative (ref)	1.24	0.77–2.02	0.37

Abbreviations: CON, control patients not treated with tamoxifen; ref, reference.

### p‐IGF‐1R/InsR and downstream PI3K/MAPK pathway activation

Expression of p‐IGF‐1R/InsR was associated with a proportionally higher number of cases scoring positive for downstream MAPK/PI3K pathway proteins and both ERα phosphorylation sites (Table 2). However, a number of p‐IGF‐1R/InsR‐negative patients might have an activated PI3K/MAPK pathway due to another upstream mechanism or crosstalk with other pathways. Conversely, p‐IGF‐1R/InsR‐positive patients may lack PI3K/MAPK pathway activation due to downstream inhibition. By using the previously described classification tool^28^ in the current setting, we identified 301 ER+, HER2‐negative patients with scores for p‐IGF‐1R/InsR and PI3K/MAPK proteins. In this group, we found a strong association between pathway activation and p‐IGF‐1R/InsR positivity (OR 6.3, 95% CI 3.6–11.1, *p* < 0.0001). One hundred thirty‐nine tumors were p‐IGF‐1R/InsR negative and were also classified as tumors without PI3K/MAPK pathway activation and 75 p‐IGF‐1R/InsR‐positive tumors had indeed pathway activation according to the classification tool.

Interestingly, results of the classification tool and p‐IGF‐1R/InsR status were discordant in 87 tumors (29%), which might affect tamoxifen outcome. In 59 ER+, HER2‐negative, p‐IGF‐1R/InsR‐negative patients in which PI3K/MAPK pathway activation was detected, tamoxifen appeared to have no effect on RFI (HR 1.09, 95% CI 0.22–5.3, *p* = 0.92). In contrast, in p‐IGF‐1R/InsR‐positive, ER+, HER2‐negative patients without pathway activation (*n* = 28) tamoxifen had a positive effect on RFI, although not statistically significant (HR 0.37, 95% CI 0.033–4.2, *p* = 0.42).

### Association of p‐ERα(Ser118) and p‐ERα(Ser167) with clinicopathological characteristics, protein expression, tamoxifen benefit and prognosis

In the group of 432 patients that could be scored for p‐ERα(Ser118), 109 RFI events occurred. The median follow‐up of patients without an event was 8.4 years (95% CI 8.1–8.9). For 468 patients with a scoring for p‐ERα(Ser167), 112 RFI events occurred with a median follow‐up of patients without an event of 8.3 years (95% CI 7.9–8.7). Patients with tumors expressing high p‐ERα(Ser118) levels had more often lymph node‐negative disease (Table S3). High p‐ERα(Ser167) tumor levels were preferentially of a lower T stage and more often HER2 negative (Table S4). High scorings of both ERα phosphorylation sites were found in a proportionally higher number of cases scoring positive for downstream MAPK/PI3K pathway proteins (Table S3 and S4).

There was no significant interaction between tamoxifen efficacy and ERα phosphorylation status (p‐ERα[Ser118]: multivariate *p* for interaction = 0.51; p‐ERα[Ser167]: multivariate *p* for interaction = 0.81; Tables S5 and S6, respectively). Interestingly, patients with high p‐ERα(Ser118) expression had no clear benefit from treatment (multivariate HR 0.72, 95% CI 0.29–1.8, *p* = 0.48), while low p‐ERα(Ser118) was associated with tamoxifen benefit (multivariate HR 0.50, 95% CI 0.27–0.93, *p* = 0.028; Fig. S4 and Table S5). Results were similar for p‐ERα(Ser167); patients with tumors highly positive for p‐ERα(Ser167) had no clear tamoxifen benefit (multivariate HR 0.58, 95% CI 0.23–1.65, *p* = 0.32), while low p‐ERα(Ser167) was associated with significant benefit (multivariate HR 0.51, 95% CI 0.29–0.88, *p* = 0.016; Fig. S5 and Table S6).

There was no significant association of p‐ERα(Ser118) (multivariate HR = 0.68, 95% CI 0.26–1.76, *p =* 0.43) or p‐ERα(Ser167) expression (multivariate HR = 0.62, 95% CI 0.23–1.65, *p =* 0.34) with RFI (Tables S5 and S6, respectively). Performing the above analyses with a cutoff of 10% for ER and PR did not substantially change the results (data not shown).

### IGF‐1R pathway activation induces PI3K/MAPK and ERα phosphorylation in human breast cancer cell lines

**Figure 3:**
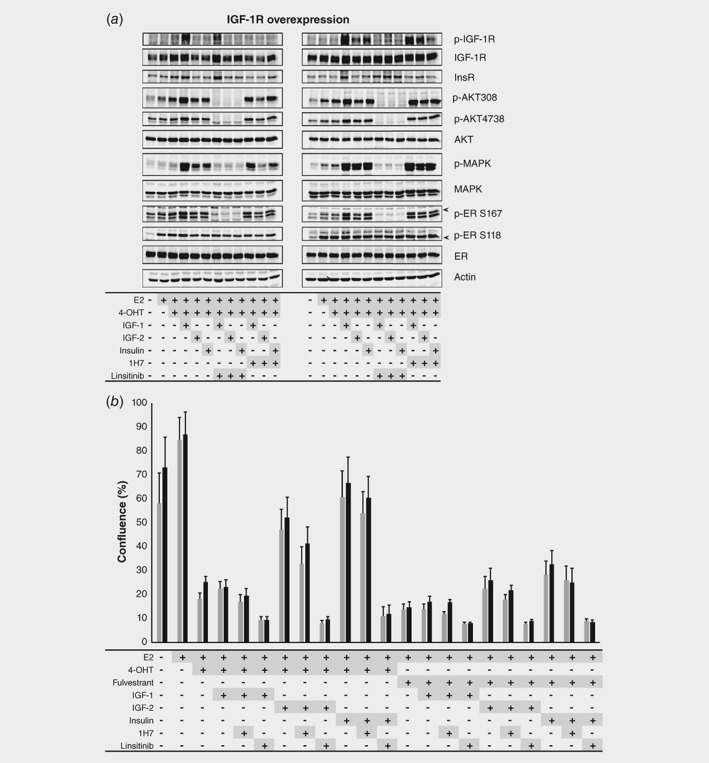
Linsitinib is able to block IGF‐1R pathway signaling and reverse tamoxifen resistance in MCF7 cells. (*a*) Representative Western blots of MCF‐7 cells without (left) and with (right) IGF‐1R overexpression showing increased (phospho‐)protein expression when cells are stimulated with growth factors and decreased expression after exposure to the dual IGF‐1R/IR inhibitor linsitinib. (*b*) IncuCyte® proliferation experiments of MCF‐7 cells without (gray bars) and with (black bars) IGF‐1R overexpression. Activation of the IGF‐1R or IR pathway by IGF‐2 or insulin restores proliferation in both cell lines pretreated with tamoxifen (4‐OHT) and to a lesser extent in case of fulvestrant exposure. Linsitinib is able to block proliferation under all conditions. Ins R, insulin receptor; E2, estrogen; 4‐OHT, tamoxifen; IGF, insuline‐like growth factor.

Preclinical data implicate IGF‐1R overexpression and its phosphorylation status to drive PI3K/MAPK pathway signaling and tamoxifen resistance,^22^ but it remains elusive whether this is due to overexpression *per se* or to activation of downstream proteins. In the absence of growth factor stimulation in IGF‐1R‐overexpressing MCF‐7 cells and empty vector control cells, the activation status of the downstream signaling cascade was similar as indicated by p‐Akt(Thr308) and p‐MAPK (Fig. 3 *a*). Phosphorylation of p‐Akt(Thr308), p‐Akt(Ser473), p‐MAPK and p‐ERα(Ser167) increased upon the addition of growth factors (IGF‐1, IGF‐2 or insulin), with p‐MAPK showing the most striking effect. This is in line with the clinical data indicating that downstream proteins were proportionally activated in tumors with positive staining for p‐IGF‐1R/InsR as compared to tumors in which p‐IGF‐1R/InsR was negative (Table 2). Interestingly, p‐ERα(Ser118) was not affected by growth factor addition, which was in contrast to an increase of p‐ERα(Ser167) upon IGR‐1R stimulation. Pretreatment with the dual IGF‐1R/InsR inhibitor linsitinib fully abrogated the activation of PI3K/MAPK signaling cascades (Fig. 3 *a* and S5A), which was accompanied by an extensive decrease in p‐ERα(Ser167). The IGF‐1R‐selective antibody 1H7 did not inhibit downstream pathway phosphorylation. Cumulatively, these data illustrate that IGF‐1R phosphorylation, but not its total protein expression level, drive the activation of downstream signaling cascades. These data were confirmed in a second ER+ breast cancer cell line, T47D (Fig. S6*a*).

### IGF‐1R activation induces ERα‐dependent tamoxifen resistance, which can be blocked by linsitinib

As described earlier, IGF‐1R phosphorylation rather than overexpression stimulates PI3K/MAPK pathway signaling ultimately increasing p‐ERα(Ser167) levels. Next, we tested whether this would affect the proliferation potential of ER+ breast cancer cells. ERα‐dependent MCF‐7 cells overexpressing IGF‐1R and empty vector control cells were hormone deprived for 3 days. Subsequently, cells were treated with IGF‐1, IGF‐2 or insulin in the presence of estradiol and either tamoxifen or fulvestrant. As expected, tamoxifen fully blocked ERα‐driven cell proliferation, but activation of the IGF‐1R pathway by IGF‐2 or insulin stimulated MCF‐7 cell proliferation (Fig. 3 *b*). Fulvestrant blocked ERα‐driven cell proliferation, irrespective of IGF‐1R activation status. Treating cells with linsitinib completely eradicated cell growth stimulation under all conditions tested, suggesting that IGF‐1R signaling is ERα‐dependent and that IGF‐1R is a factor involved in endocrine therapy resistance. In line with the phosphoprotein data, 1H7 was not able to block cell proliferation (Fig. 3 *b*). Cumulatively, these data indicate that ERα is functionally and critically involved in the observed tamoxifen‐resistant phenotype. These results were confirmed in T47D cells (Fig. S6*b*).

## Discussion

Here, we reveal that IGF‐1R pathway activation may be disadvantageous for the efficacy of adjuvant tamoxifen in postmenopausal ER+ breast cancer patients and that blocking this activated pathway may reverse this mechanism of endocrine therapy resistance.

In our patient cohort, presence of p‐IGF‐1R/InsR was associated with a better RFI in untreated patients. IGF‐1R and its phosphorylation status have been studied as a possible prognostic biomarker in breast cancer patients. In a recent meta‐analysis, both positive membranous and cytoplasmic IGF‐1R protein levels were reported as a favorable prognostic factor for overall and breast cancer‐specific survival in patients with hormone receptor‐positive breast cancer.^33^ In the study of Bjorner *et al*.,^34^ positive p‐IGF‐1R/InsR staining was associated with a lower risk for events among non‐endocrine‐treated patients, irrespective of ER status. Overall, our results confirm that positivity of p‐IGF‐1R/InsR might serve as a marker for better prognosis in ER+ primary breast cancer.

We showed in patients with IGF‐1R‐positive tumors that those with p‐IGF‐1R/InsR expression derived no clear benefit from adjuvant tamoxifen, while patients without p‐IGF‐1R/InsR positivity had an improved RFI on tamoxifen. The pivotal test for interaction was not significant, which is possibly due to the relatively small number of patients and specifically the lack of sufficient control patients. Another explanation might be that some p‐IGF‐1R/InsR‐negative patients still had an activated PI3K/MAPK pathway, for instance due to crosstalk with other pathways and, therefore, the effect of tamoxifen on outcome was reduced. When we applied our recently published classification tool^28^ to the current setting to demonstrate PI3K/MAPK pathway activation, we indeed found some p‐IGF‐1R/InsR‐negative patients with PI3K/MAPK pathway activation. Although this might explain our results, additional validation studies are required.

Based on preclinical observations that PI3K/MAPK pathway activation can induce phosphorylation at both ERα sites,^14^, ^15^ we investigated whether ERα(Ser118) or ERα(Ser167) phosphorylation status was associated with PI3K/MAPK pathway activation and tamoxifen outcome in our patients. High p‐ERα(Ser118) or p‐ERα(Ser167) expression was associated with phosphorylation of proteins within the PI3K/MAPK pathway and absence of tamoxifen benefit, although the interaction test for tamoxifen benefit was not significant. Studies on the role of p‐ERα(Ser118) in breast cancer patients treated with adjuvant tamoxifen were conflicting as some groups presented negative results,^35^, ^36^, ^37^ while others described that p‐ERα(Ser118) might predict response to endocrine therapy.^17^, ^38^ Expression of p‐ERα(Ser167) in primary tumors was predictive for response to endocrine therapy in the metastatic setting as reported by Yamashita *et al*. 37 Studies other than ours demonstrating an effect of p‐ERα(Ser167) on adjuvant endocrine therapy responses in patients are lacking. Based on the aggregated data, measuring ER phosphorylation status as a predictive marker for absence of tamoxifen benefit is not yet ready for implementation in daily clinical practice.

Since the clinical finding of IGF‐1R pathway activation might be a factor for adjuvant tamoxifen failure, we performed functional studies in ER+ breast cancer cell lines to test this hypothesis in a controlled setting. Indeed, in ERα‐positive breast cancer cell lines, IGF‐1R activation rather than IGF‐1R (over)expression stimulated downstream MAPK/PI3K signaling cascades, resulting in cell proliferation despite tamoxifen exposure. Massarweh *et al*.^24^ have also observed that IGF‐1R activation rather than overexpression contributed to tamoxifen resistance. In their study, tamoxifen‐resistant MCF7 xenografts had increased levels of p‐IGF‐1R compared to tamoxifen‐sensitive xenografts. These results support the clinical observation that IGF‐1R activation, rather than its overexpression, confers endocrine resistance.

Our cell line experiments showed that stimulation of IGF‐1R enhanced p‐ERα(Ser167), but not p‐ERα(Ser118) in MCF7 cells. The direct impact of IGF‐1R activation on ERα phosphorylation at Serine 118 and Serine 167 has been well established.^15^ ERα(Ser118) can be phosphorylated by a number of kinases, including CDK7 and MAPK,^39^, ^40^ while p70S6K, Akt and MAPK are known to phosphorylate ERα(Ser167).^15^, ^41^ Our results suggest that linsitinib affects ERα(Ser167) phosphorylation in both the MCF7 and T47D cell line, while linsitinib diminishes ERα(Ser118) phosphorylation only in T47D cells. Since proliferation was successfully inhibited in both cell lines by linsitinib, it appears that ERα(Ser167) phosphorylation might be a more relevant IGF‐1R‐dependent phosphorylation site on ER to drive tumor cell proliferation capacity than phosphorylation of ERα(Ser118).

A randomized Phase 2 study in hormone receptor‐positive, HER2‐negative, metastatic breast cancer patients combining endocrine therapy with placebo or ganitumab, a monoclonal antibody directed against IGF‐1R, turned out negative.^42^ Our cell line data may give an explanation for this result. In the ER+ breast cancer cell lines, a specific antibody against IGF‐1R was not able to block downstream PI3K/MAPK signaling or tumor cell proliferation, unlike the dual IGF‐1R/InsR inhibitor linsitinib. This suggests that binding to IGF‐1R alone is not sufficient for a good antitumor response and that in tumor cells the PI3K/MAPK pathway may remain activated, possibly also through InsR. Previous preclinical research underpins our findings that inhibition of both IGF‐1R and InsR is necessary for a good antitumor response.^43^ Future research should be performed to elucidate the potential impact of InsR as well as InsR/IGF1R crosstalk in the observed tamoxifen resistance phenotype.

The potential clinical applicability of linsitinib has been studied in a variety of advanced cancer patients, but these trials failed to demonstrate antitumor activity.^44^, ^45^, ^46^ Our data suggest that the subset of high‐risk postmenopausal ER+ breast cancer patients characterized by PI3K/MAPK pathway activation *via* IGF‐1R may have more benefit from adjuvant tamoxifen to which linsitinib is added than from adjuvant tamoxifen alone. Naturally, the latter should be validated in an independent prospective randomized clinical trial.^47^

Currently, postmenopausal ER+ breast cancer patients generally receive tamoxifen in sequence with an AI, or 5 years of an AI as adjuvant endocrine therapy for at least 5 years.^3^ Patients in our retrospective study received a maximum of 3 years of tamoxifen and no additional AI. Although others have described that PI3K pathway activation is associated with resistance irrespective of the type of endocrine treatment,^7^ it is unknown whether activation of the IGF‐1R pathway would also lead to less benefit from an AI. Furthermore, whether our results can be applied to other patients on treatment for five or more years is not known. It is highly unlikely, however, that activation of the PI3K/MAPK pathways in tumor cells as an intrinsic resistance mechanism will be lost after longer endocrine treatment duration. Nevertheless, future studies should clarify whether our findings can be extrapolated to current treatment regimens.

In conclusion, our study demonstrates that stimulation of IGF‐1R activates the PI3K and MAPK pathways in ER+ breast cancer cell lines, contributing to tamoxifen resistance. This observation is supported by the lack of benefit from adjuvant tamoxifen in postmenopausal ER+, IGF‐1R‐positive breast cancer patients with tumors staining positive for p‐IGF‐1R/InsR. We also showed that exposing breast cancer cells to the dual IGF1R/InsR inhibitor linsitinib can abrogate IGF‐1R signaling and restore endocrine therapy sensitivity. Even though our findings require validation in an independent cohort of ER+, IGF‐1R‐positive breast cancer patients on adjuvant tamoxifen, our data suggest that IGF‐1R/InsR inhibition might be an overlooked treatment option for patients with tumors harboring an activated IGF‐1R signaling route.

## Supporting information


**Appendix S1**: Supplementary Material

## Data Availability

The data that support the findings of our study are available from the corresponding author upon reasonable request.
