# Supplementary material for: IGF‐1R pathway activation as putative biomarker for linsitinib therapy to revert tamoxifen resistance in ER‐positive breast cancer
Source: Int J Cancer. 2019 Oct 6;146(8):2348–59. doi: 10.1002/ijc.32668 (PMC7065127; doi:10.1002/ijc.32668)
Supplement: Supplementary file 1 — Appendix S1: Supplementary Material [file IJC-146-2348-s001.doc]

# IGF-1R pathway activation as putative biomarker for linsitinib therapy to revert tamoxifen resistance in ER-positive breast cancer

**Running title: IGF-1R pathway activation and tamoxifen resistance**

Dinja T Kruger, Xanthippi Alexi, Mark Opdam, Karianne G Schuurman, Leonie Voorwerk, Joyce Sanders, Vincent van der Noort, Epie Boven, Wilbert Zwart, Sabine C Linn

# Supplements

**Supplementary Tables**

## Table S1

Immunohistochemistry: antibodies, scoring procedures and kappa coefficients to determine the inter-observer variability

| **Antibody** | **Article Number (clone)** | **Scoring system** | **Cutoff for binary score** | **Number of patients used for kappa calculation** | **Kappa for binary score** |
| --- | --- | --- | --- | --- | --- |
| IGF-1R | Ventana Medical Systems  #790-4346 (G11) | Membranous intensity | 0 vs 1-3 | 107 | 0.71 |
| p-IGF-1R/IR  (Tyr1131/Tyr1146))* | Cell Signaling  # 3021 | Cytoplasmic intensity | 0 vs 1-3 | 160 | 0.82 |
| p-ERα(Ser118) | Cell Signaling  #2511 | Proportion of tumor cells with nuclear staining | <50% vs ≥50% | 95 | 0.61 |
| P-ERα(Ser167) | Cell Signaling  #5587 | Proportion of tumor cells with nuclear staining | <50% vs ≥50% | 61 | 0.86 |
| PTEN | Cell Signaling  #9559 (138G6) | Cytoplasmic intensity | 0 *vs* 1-3 | 126 | 0.75 |
| p-Akt(Thr308) | Cell Signaling  #2965 (C31E5E) | Cytoplasmic intensity | 0 *vs* 1-3 | 133 | 0.53 |
| p-Akt(Ser473) | Cell Signaling  #4060 (D9E) | Cytoplasmic intensity | 0-1 *vs* 2-3 | 94 | 0.70 |
| p-4EBP1(Ser65) | Cell Signaling  #9456 (174A9) | Percentage of tumor cells with nuclear staining | 0-50 *vs* 51-100 | 112 | 0.59 |
| p-p70S6K (Thr389) | Cell Signaling  #9206 (1A5) | Cytoplasmic intensity | 0 *vs* 1-3 | 97 | 0.44 |
| p-p44/42 MAPK(ERK1/2) (Thr202/Tyr204) | Cell Signaling  #4370 (D13.14.4E) | Proportion of tumor cells with nuclear staining | 0% *vs* ≥1% | 69 | 0.85 |
| p-S6RP (Ser235/236) | Cell Signaling  #2211 | Percentage of tumor cells with cytoplasmic and membranous staining | 0-19 *vs* 20-100 | 85 | 0.55 |

*antibody detects both p-IGF-1R(Tyr1131) and p-IR(Tyr1146)

## Table S2

Western Blot: antibodies and dilutions

| **Antibody** | **Article Number (clone)** | **Dilution Western blot** |
| --- | --- | --- |
| IGF-1R | Ventana Medical Systems #790-4346 (G11) | 1:250 |
| p-IGF-1R/IR(Tyr1135/1136)* | Cell Signaling #3024 (19H7) | 1:500 |
| Insulin receptor β | Cell Signaling #3025 (4B8) | 1:1000 |
| p-ERα(Ser118) | Cell Signaling #2511 (16J4) | 1:1000 |
| P-ERα(Ser167) | Cell Signaling #5587 (D1A3) | 1:1000 |
| Estrogen receptor α | Thermo Fisher #MA5-14104 (1D5+6F11) | 1:500 |
| p-Akt(Thr308) | Cell Signaling #2965 (C31E5E) | 1:500 |
| p-Akt(Ser473) | Cell Signaling #9277 | 1:1000 |
| Akt1 | Cell Signaling #2938 (C73H10) | 1:1000 |
| p-p44/42 MAPK(ERK1/2) (Thr202/Tyr204) | Cell Signaling #4370 (D13.14.4E) | 1:500 |
| p44/42 MAPK(ERK1/2) | Cell Signaling #9102 | 1:1000 |
| Actin | Millipore Merck MAB1501R (C4) | 1:10000 |

*antibody detects both p-IGF-1R(Tyr1135/1136) and p-IR(Tyr1150/1151)

## Table S3

Association between clinicopathological factors and downstream proteins in high vs low p-ERα(Ser118)

|  |  |  | **p-ERα(Ser118)** |  |
| --- | --- | --- | --- | --- |
|  |  | < 50% | ≥ 50% |  |
|  |  | N (%) | N (%) | p-value* |
| Age | <65 | 122 (47) | 88 (50) | 0.62 |
|  | ≥65 | 135 (53) | 87 (50) |  |
| Lymph node status | Negative | 122 (47) | 104 (59) | 0.018 |
|  | Positive | 135 (53) | 71 (41) |  |
| T stage | T1-2 | 223 (87) | 161 (92) | 0.12 |
|  | T3-4 | 34 (13) | 14 (8) |  |
| Grade | Grade 1-2 | 149 (58) | 117 (67) | 0.07 |
|  | Grade 3 | 108 (42) | 58 (33) |  |
| Histological subtype | Ductal | 197 (90) | 127 (89) | 0.86 |
|  | Lobular | 21 (10) | 15 (11) |  |
| HER2 status | Negative | 224 (89) | 161 (93) | 0.23 |
|  | Positive | 27 (11) | 12 (7) |  |
| PR status | Negative | 106 (41) | 58 (33) | 0.11 |
|  | Positive | 150 (59) | 116 (67) |  |
| PTEN | 0 | 65 (27) | 11 (6) | <0.0001 |
|  | 1-3 | 178 (73) | 159 (94) |  |
| p-Akt(Thr308) | 0 | 170 (70) | 59 (35) | <0.0001 |
|  | 1-3 | 73 (30) | 110 (65) |  |
| p-Akt(Ser473) | 0-1 | 127 (59) | 33 (21) | <0.0001 |
|  | 2-3 | 90 (41) | 128 (79) |  |
| p-4EBP1 | 0-20% | 130 (56) | 33 (21) | <0.0001 |
|  | 30-100% | 104 (44) | 129 (79) |  |
| p-p70S6K | 0 | 150 (61) | 27 (16) | <0.0001 |
|  | 1-3 | 97 (39) | 142 (84) |  |
| p-MAPK | 0% | 148 (62) | 29 (17) | <0.0001 |
|  | 10 – 100% | 91 (38) | 142 (83) |  |
| p-S6RP | 0 – 10% | 98 (41) | 35 (22) | <0.0001 |
|  | 20 – 100% | 140 (59) | 127 (78) |  |
| p-ERα(Ser167) | 0-40% | 227 (92) | 67 (40) | <0.0001 |
|  | 50-100% | 20 (8) | 102 (60) |  |

* Fisher’s exact test based on cases without missing values

## Table S4

Association between clinicopathological factors and downstream proteins in high vs low p-ERα(Ser167)

|  |  |  | **p-ERα(Ser167)** |  |
| --- | --- | --- | --- | --- |
|  |  | < 50% | ≥ 50% |  |
|  |  | N (%) | N (%) | p-value* |
| Age | <65 | 154 (46) | 64 (48) | 0.61 |
|  | ≥65 | 182 (54) | 68 (52) |  |
| Lymph node status | Negative | 175 (52) | 80 (61) | 0.1 |
|  | Positive | 161 (48) | 52 (39) |  |
| T stage | T1-2 | 290 (86) | 123 (93) | 0.039 |
|  | T3-4 | 46 (14) | 9 (7) |  |
| Grade | Grade 1-2 | 207 (62) | 95 (72) | 0.041 |
|  | Grade 3 | 129 (38) | 37 (28) |  |
| Histological subtype | Ductal | 245 (88) | 101 (94) | 0.14 |
|  | Lobular | 34 (12) | 7 (6) |  |
| HER2 status | Negative | 291 (89) | 123 (96) | 0.026 |
|  | Positive | 35 (11) | 5 (4) |  |
| PR status | Negative | 129 (39) | 47 (36) | 0.6 |
|  | Positive | 202 (61) | 84 (64) |  |
| PTEN | 0 | 64 (22) | 11 (9) | 0.0029 |
|  | 1-3 | 231 (78) | 108 (91) |  |
| p-Akt(Thr308) | 0 | 210 (66) | 42 (34) | <0.0001 |
|  | 1-3 | 110 (34) | 83 (66) |  |
| p-Akt(Ser473) | 0-1 | 144 (53) | 20 (18) | <0.0001 |
|  | 2-3 | 128 (47) | 92 (82) |  |
| p-4EBP1 | 0-20% | 145 (48) | 26 (21) | <0.0001 |
|  | 30-100% | 158 (52) | 97 (79) |  |
| p-p70S6K | 0 | 160 (53) | 18 (15) | <0.0001 |
|  | 1-3 | 142 (47) | 106 (85) |  |
| p-MAPK | 0% | 164 (54) | 17 (14) | <0.0001 |
|  | 10 – 100% | 139 (46) | 108 (86) |  |
| p-S6RP | 0 – 10% | 131 (42) | 21 (17) | <0.0001 |
|  | 20 – 100% | 178 (58) | 101 (83) |  |
| p-ERα(Ser118) | 0-40% | 227 (77) | 20 (16) | <0.0001 |
|  | 50-100% | 67 (23) | 102 (84) |  |

* Fisher’s exact test based on cases without missing values

## Table S5

Multivariate Cox proportional hazard model of recurrence-free interval including p-ERα(Ser118) status and treatment interaction

| **Variable** |  | **HR** | **95% CI** | ***p*** |
| --- | --- | --- | --- | --- |
| Interaction | p-ERα(Ser118) with treatment |  |  | 0.51 |
| Tamoxifen vs CON | p-ERα(Ser118) < 50% group | 0.50 | 0.27 – 0.93 | 0.028 |
|  | p-ERα(Ser118) ≥ 50% group | 0.72 | 0.29 – 1.80 | 0.48 |
| p-ERα(Ser118) ≥ 50% vs < 50% | CON patients | 0.68 | 0.26 – 1.76 | 0.43 |
| Age | ≥65 vs <65 (ref) | 1.05 | 0.69 – 1.61 | 0.80 |
| T stage | T3-4 vs T1-2 (ref) | 1.61 | 0.92 – 2.81 | 0.09 |
| Grade | Grade 3 vs grade 1-2 (ref) | 1.50 | 0.93 – 2.39 | 0.091 |
| Histological subtype | Lobular vs ductal (ref) | 2.00 | 1.05 – 3.81 | 0.035 |
| HER2 status | Positive vs negative (ref) | 1.40 | 0.74 – 2.65 | 0.31 |
| PR status | Positive vs negative (ref) | 1.13 | 0.73 – 1.75 | 0.59 |

HR: hazard ratio; CI: confidence interval; CON: control patients not treated with tamoxifen; ref: reference

## Table S6

Multivariate Cox proportional hazard model of recurrence free interval (RFI) including p-ERα(Ser167) status and treatment interaction

| **Variable** |  | **HR** | **95% CI** | ***p*** |
| --- | --- | --- | --- | --- |
| Interaction | p-ERα(Ser167) with treatment |  |  | 0.81 |
| Tamoxifen vs CON | p-ERα(Ser167) < 50% group | 0.51 | 0.29 – 0.88 | 0.016 |
|  | p-ERα(Ser167) ≥ 50% group | 0.58 | 0.20 – 1.68 | 0.329 |
| p-ERα(Ser167) ≥ 50% vs < 50% | CON patients | 0.62 | 0.23 – 1.65 | 0.34 |
| Age | ≥65 vs <65 (ref) | 1.00 | 0.65 – 1.51 | 0.98 |
| T stage | T3-4 vs T1-2 (ref) | 1.61 | 0.94 – 2.74 | 0.081 |
| Grade | Grade 3 vs grade 1-2 (ref) | 1.49 | 0.94 – 2.38 | 0.092 |
| Histological subtype | Lobular vs ductal (ref) | 2.07 | 1.12 – 3.83 | 0.021 |
| HER2 status | Positive vs negative (ref) | 1.31 | 0.68 – 2.54 | 0.42 |
| PR status | Positive vs negative (ref) | 1.15 | 0.74 – 1.79 | 0.52 |

HR: hazard ratio; CI: confidence interval; CON: control patients not treated with tamoxifen; ref: reference

**Supplementary figures**

## Figure S1. Correlation plots between IGF-1R, membranous p-IGF-1R/IR and cytoplasmic p-IGF-1R/IR


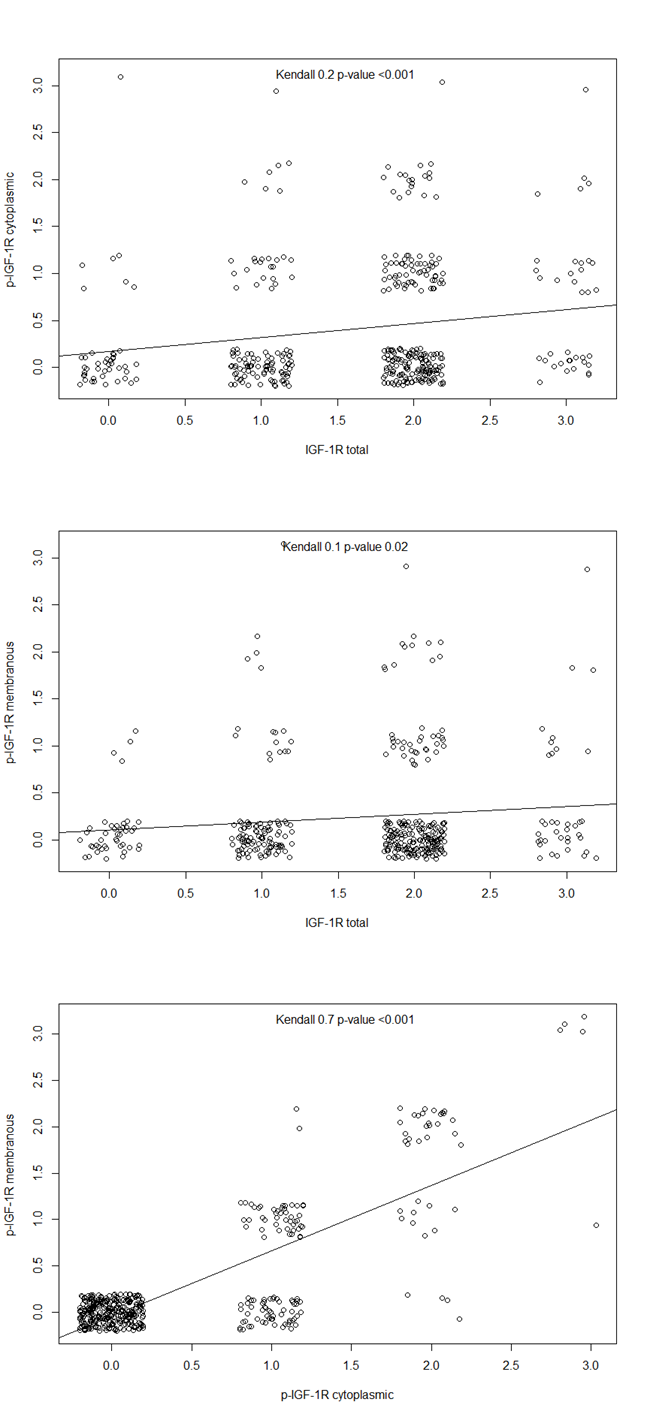


## Figure S2. Distribution of staining scores for p-ERα(Ser118) and p-ERα(Ser167) in ER+ patients.


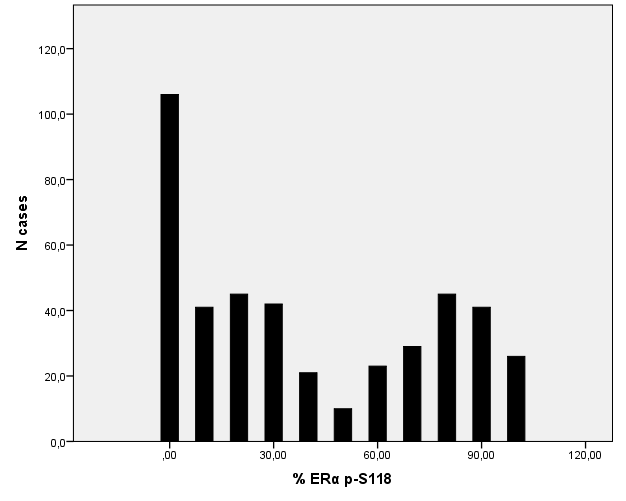


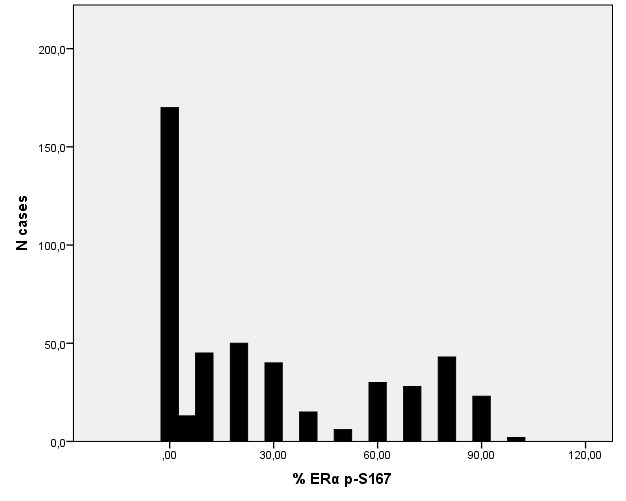


## Figure S3. Western blot with a subset of samples used to quantify IGF-1R.

**
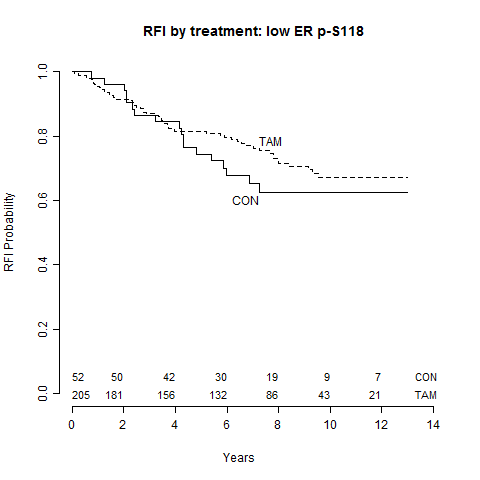

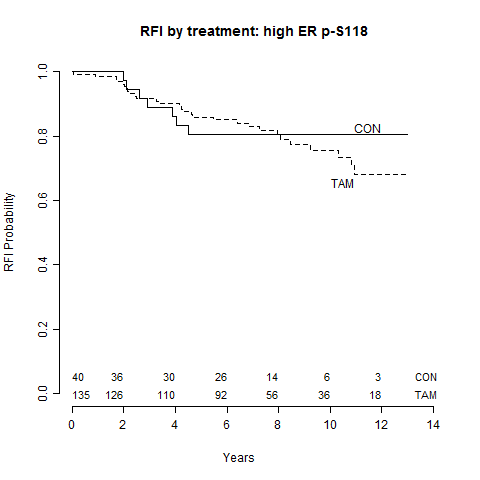
**

**A**

**B**

Multivariate HR 0.50, *p* = 0.028

Multivariate HR 0.72, *p* = 0.48

## Figure S4. p-ERα(Ser118) and tamoxifen benefit

1. Kaplan-Meier curves for recurrence-free interval according to tamoxifen treatment in patients with low p-ERα(Ser118) expression in their tumor tissue
2. Kaplan-Meier curves for recurrence-free interval according to tamoxifen treatment in patients with high p-ERα(Ser118) expression in tumor tissue.

TAM: patients treated with tamoxifen; CON: control patients not treated with tamoxifen. Multivariate *p* for interaction = 0.51.


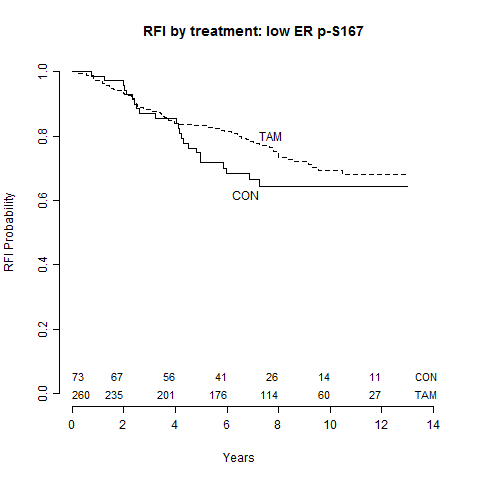

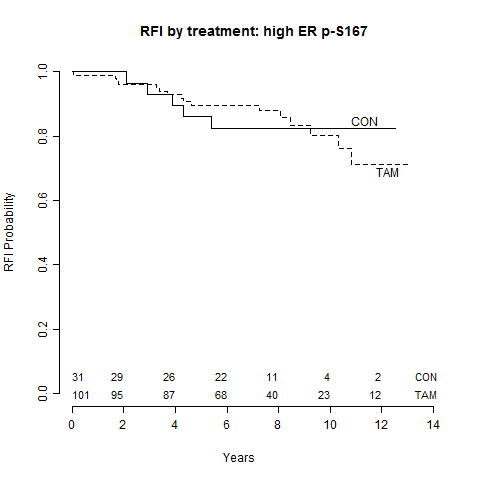


**B**

**A**

Multivariate HR 0.51, *p* = 0.016

Multivariate HR 0.58, *p* = 0.32

## Figure S5. p-ERα(Ser167) and tamoxifen benefit

1. Kaplan-Meier curves for recurrence-free interval according to tamoxifen treatment in patients with low p-ERα(Ser167) expression in their tumor tissue
2. Kaplan-Meier curves for recurrence-free interval according to tamoxifen treatment in patients with high p-ERα(Ser167) expression in tumor tissue.

TAM: patients treated with tamoxifen; CON: control patients not treated with tamoxifen. Multivariate *p* for interaction = 0.81.


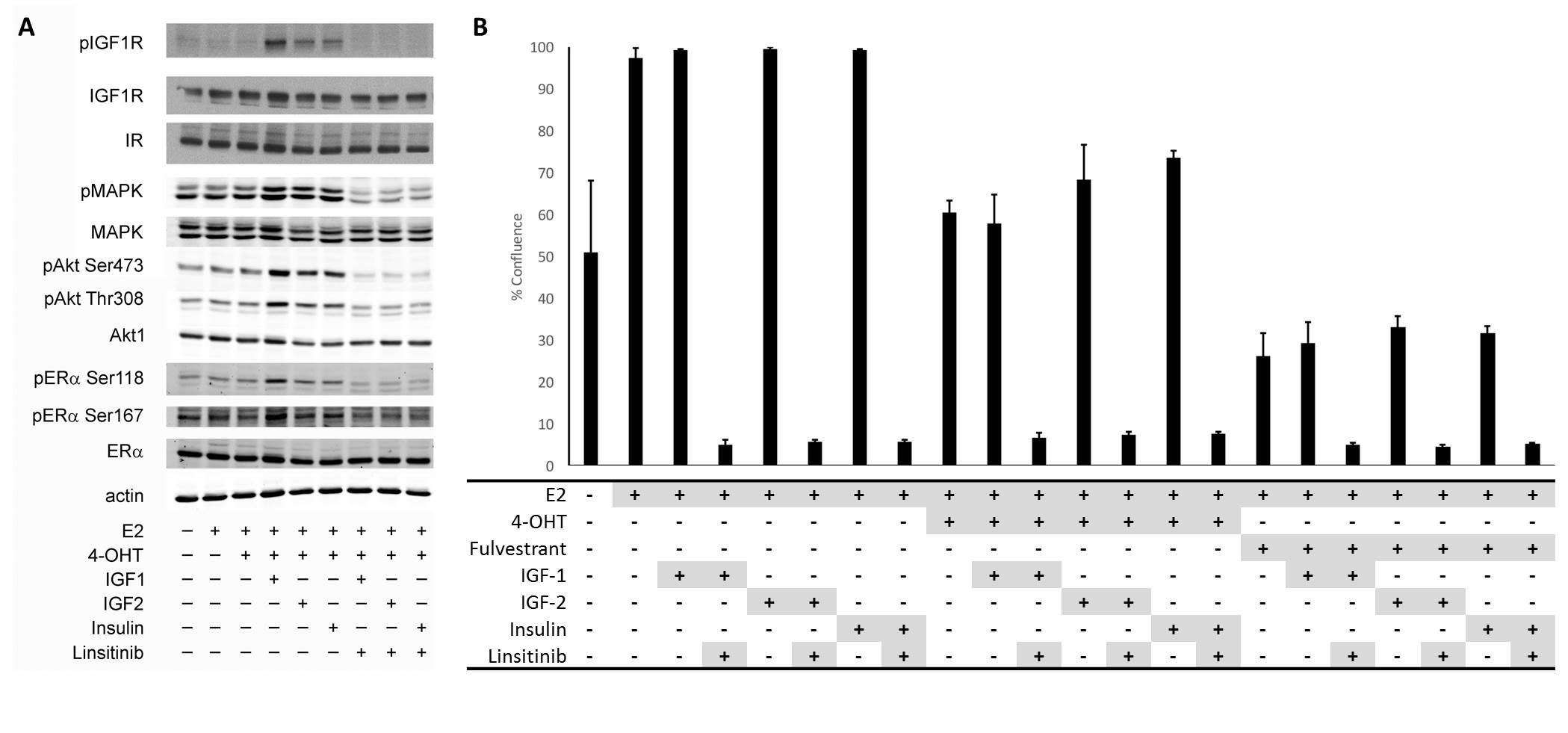


## Figure S6. Western blot and IncuCyte® proliferation experiments in T47D cells

1. Representative Western blot of T47D cells showing increased (phospho)protein expression when cells were stimulated with IGF-1, IGF-2 or insulin and decreased expression after exposure to the dual IGF-1R/IR inhibitor linsitinib.
2. IncuCyte® proliferation experiments of T47D cells. Activation of the IGF-1R or IR pathway by IGF-1, IGF-2 or insulin restored proliferation in both cell lines pretreated with tamoxifen (4-OHT) and to a lesser extent in case of fulvestrant exposure. Linsitinib is able to block proliferation under all conditions.

IR: insuline receptor; E2: estrogen; 4-OHT: tamoxifen; IGF: insuline-like growth factor.
